# The COMET Initiative database: progress and activities update (2015)

**DOI:** 10.1186/s13063-017-1788-8

**Published:** 2017-02-03

**Authors:** E. Gargon, P. R. Williamson, D. G. Altman, J. M. Blazeby, S. Tunis, M. Clarke

**Affiliations:** 1 Department of Biostatistics, University of Liverpool, 1st floor Duncan Building, Daulby Street, Liverpool, L69 3GA UK; 2 University of Oxford, Centre for Statistics in Medicine, Botnar Research Centre, Windmill Road, Oxford, OX3 7LD UK; 3 School of Social and Community Medicine, University of Bristol, Canynge Hall, 39 Whatley Road, Bristol, BS8 2PS UK; 4 Center for Medical Technology Policy (CMTP), World Trade Center Baltimore, 401 East Pratt Street, Suite 631, Baltimore, MD 21202 USA; 5 Queen’s University Belfast, Institute of Clinical Sciences, Block B, Royal Hospitals, Grosvenor Road, Belfast, BT12 6BJ UK

**Keywords:** Core outcome set, Database, Resources

## Abstract

This letter describes the substantial activity on the Core Outcome Measure in Effectiveness Trials (COMET) website in 2015, updating our earlier progress reports for the period from the launch of the COMET website and database in August 2011 to December 2014. As in previous years, 2015 saw further increases in the annual number of visits to the website, the number of pages viewed and the number of searches undertaken. The sustained growth in use of the website and database suggests that COMET is continuing to gain interest and prominence, and that the resources are useful to people interested in the development of core outcome sets.

## Correspondence/findings

### Background

As the New Year bells were ringing and the fireworks were exploding to welcome 2016 in cities such as Fray Bentos in South America, an Internet user in Tianjin, China ran the 10,000th search of the Core Outcome Measures in Effectiveness Trials (COMET) database. They were looking for information about non-small-cell lung cancer (NSCLC), and will have been shown details of two core outcome sets (COS) recently added to the database [1, 2]. This letter describes the substantial activity on the COMET website [3] in the 365 days before that search. We update our earlier progress since the launch of the COMET website and database in August 2011 to December 2014 [4, 5].

### Activity and content

A total of 720 studies relevant to the development of COS were included in the COMET database at the end of December 2015, with 147 added during the year. This included 32 reports relating to 29 COS identified in the most recent update to the systematic review of COS [2], which had originally been performed in 2013 [1].

**Fig. 1:**
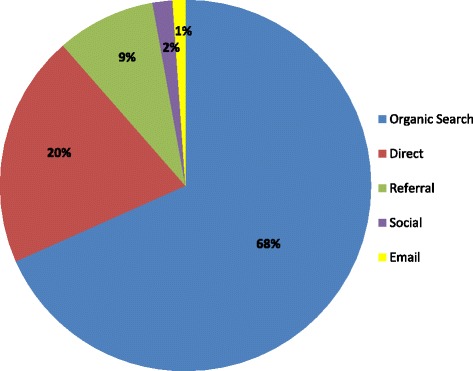
Core Outcome Measures in Effectiveness Trials (COMET) website acquisition overview

As in previous years, 2015 saw further increases in the annual number of visits to the website (Table 1) and although the proportional change declined in 2015, the absolute numbers continue to increase. For instance, the proportional increase in new visitors from 2014 to 2015 was 33%, compared to 43% for 2013 to 2014; but the absolute increase from 2014 to 2015 was 3269 compared to 2936 for 2013 to 2014. Most visitors to the website arrived either via links following an organic search using a search engine, such as Google (68%), or direct (20%) (Fig. 1). However, new for 2015, were arrivals from links in emails, which is in large part attributable to the move of the COMET newsletter from a PDF format to an email format.

**Table 1:** Core Outcome Measures in Effectiveness Trials (COMET) website usage statistics 2012 to 2015

	Number of visits				Number of unique visitors				Number of new visitors				Number of searches			
Year	2012	2013	2014	2015	2012	2013	2014	2015	2012	2013	2014	2015	2012	2013	2014	2015
Total	7982	12332	16768	20952	5471	8369	12257	15366	4611	6844	9780	13049	1597	2139	2383	3411
Increase per year (%)	n/a	55%	36%	25%	n/a	53%	47%	25%	n/a	48%	43%	33%	n/a	34%	11%	43%
Overall increase from 2012 to 2015 (%)	163%				181%				183%				114%

Social media also leads many people to the website and Twitter accounted for 89% of social referrals to the COMET website in 2015 (Table 2). The COMET Twitter account is monitored by the research team and tweets are sent when new COS papers are published, to announce relevant presentations at conferences and to retweet COS-related tweets from others that we follow. The COMET account has more than 1300 followers and the Twitter page links to the COMET website.

**Table 2:**
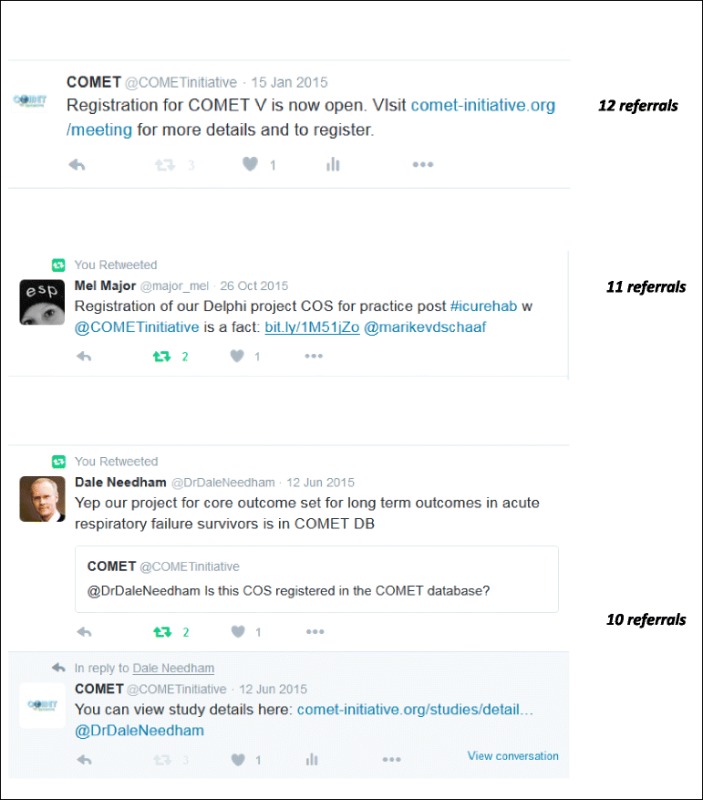
Examples of Twitter referrals

The highest proportions of referrals were from the Core Outcomes in Women’s Health (CROWN) Initiative (10%), the University of Liverpool (6%), *Trials* journal (5%), MRC Hubs for Trials Methodology Research (5%), Cochrane Canada (5%) and the Standard Protocol Items: Recommendations for Interventional Trials (SPIRIT) initiative (4%). CROWN is an international initiative to harmonise outcome reporting in women’s health research. More than 70 journals have committed to encouraging the development and reporting of COS in this area and CROWN advises all COS developers to register with COMET [6]. *Trials* has published several COS papers which generated referrals to the COMET website, including ‘Developing core outcome sets for clinical trials: issues to consider’ [7], the special collection of the meeting proceedings and abstracts from the 4th COMET meeting in Rome in November 2014, and the report of the first meeting to discuss Trial Forge [8]. The 5th COMET meeting was jointly hosted with Cochrane Canada in Calgary in May 2015, hence the large number of referrals from Cochrane Canada, and the more than doubling in the annual number of visits from Canada, from 624 in 2014 to 1449 in 2015 (Table 3). The referrals from SPIRIT reflect that initiative’s encouragement of trial investigators to consider measuring the outcomes in a COS in their trial as part of their effort to improve the quality of clinical trial protocols by defining an evidence-based set of items to address in a protocol.

**Table 3:** Countries with the most visits to the Core Outcome Measure in Effectiveness Trials (COMET) website in 2012 to 2015

2012		2013		2014		2015	
United Kingdom	5577	United Kingdom	7526	United Kingdom	8203	United Kingdom	9862
United States	431	United States	1022	United States	2038	United States	2444
Canada	326	Canada	501	Italy	1115	Canada	1449
Australia	201	Australia	321	Canada	624	Australia	654
Germany	186	Italy	315	Germany	581	France	593
Netherlands	166	Netherlands	308	Netherlands	510	Netherlands	570
Italy	161	Germany	285	Australia	494	Germany	553
France	125	Japan	228	France	374	India	477
Ireland	113	France	227	India	306	Italy	439
Norway	62	Ireland	159	Ireland	239	Ireland	415

In 2015, there were a total 80,799 page views, a 10% increase from 73,617 in 2014. Analyses of the COMET website data show that 56% of visitors went beyond the page on which they landed in 2015, similar to 2014 and, as in previous years, the most common first interaction was to search the COMET database. Other first interactions included moving to the overview of the COMET Initiative, accessing the database without completing a search, and checking the resources page. This Core Resource Pack is once again the second most highly accessed resource on the website (after the database), with 1372 page views in 2015, compared to 1064 in 2014 (29% increase).

The total number of visits increased by 25% in 2015 compared to 2014.The number of unique visitors also increased by 25%, and the number of new visitors increased by 33%. Full details are provided in Table 1. Visitors came from 127 countries, with 53% of visits now coming from outside the UK, an increase of 2% from 8565/16,768 in 2014 to 11,090/20,952 in 2015 (Table 3).

By the end of December 2015 in the time zone of the COMET website, a total of 9999 searches had been undertaken in the COMET database, with 3411 in 2015 alone (a 43% increase from 2014). The most frequently used search criteria were consistent with previous years with the most frequently searched category being Disease Category. The ‘top 10’ searched for terms are shown in Table 4. In 2014, the most commonly searched term was ‘cancer’ (*n* = 129) and although this increased to 137 searches in 2015, it was superseded in 2015 by ‘pregnancy and childbirth’ (*n* = 193).

**Table 4:** ‘Top 10’ search terms in 2015

Category	Number
Pregnancy and childbirth	193
Cancer	137
Neurology	88
Mental health	79
Gynaecology	77
Skin	76
Heart and circulation	69
Anaesthesia and pain control	68
Dentistry and oral health	65
Orthopaedics and trauma	65

In 2015, we conducted a pop-up survey to find out why people were searching in the COMET database. The survey appeared at the beginning of each search during a 1-month period, asking people to select single response to give their reason for searching in the COMET database. Full details of the survey have been published [2] but, in summary, it showed that the most common reasons for searching the database were to inform decision-making about developing a COS, or to inform the outcomes in planning a clinical trial. The pop-up survey also confirmed the importance of keeping the contents of the database up to date, if it is to help researchers to avoid unnecessary duplication of effort and minimise waste [9].

The sustained growth in use of the website and database suggests that COMET is continuing to gain interest and prominence, and that the resources are useful to people interested in COS development. To help ensure that the content is kept up to date a second update of the systematic review of COS [1, 2] is underway and the COMET website and database usage figures will continue to be monitored and assessed annually.

## Abbreviations

COMET: Core Outcome Measures in Effectiveness Trials
COS: Core outcome set(s)
CROWN: Core Outcomes in Women’s Health
NSCLC: Non-small-cell lung cancer
SPIRIT: Standard Protocol Items: Recommendations for Interventional Trials
